# Bioluminescent and Fluorescent Proteins: Molecular Mechanisms and Modern Applications

**DOI:** 10.3390/ijms24010281

**Published:** 2022-12-23

**Authors:** Eugene S. Vysotski

**Affiliations:** Photobiology Laboratory, Institute of Biophysics SB RAS, Federal Research Center “Krasnoyarsk Science Center SB RAS”, Krasnoyarsk 660036, Russia; eugene_vysotski@ibp.ru

## 1. Key Events in Bioluminescent and Fluorescent Proteins Studies

Light emission by living organisms in the visible spectrum range is called bioluminescence. Despite the fact that luminous organisms are found among terrestrial species (bacteria, fungi, worms, and fireflies), the phenomenon of bioluminescence is most common among marine creatures [1]. At depth, more than 90% of organisms are luminous [2]. This implies that bioluminescence may be of vital importance for the activity (communication, camouflage from predators, confusing predators by light, attracting prey, etc.) of host species. However, its exact significance for many luminous organisms is not yet clear.

In fact, bioluminescence is a result of the enzymatic reaction in which the enzyme, luciferase, catalyzes oxidation of the substrate, luciferin, by the molecular oxygen. The study of bioluminescent reactions of organisms belonging to different taxa has revealed an amazing variety of mechanisms underlying light emission and the substrates and cofactors involved in the reactions, as well as the enzymes catalyzing these reactions. Thus, the terms “luciferase” and “luciferin” used to denote enzymes and substrates of bioluminescent reactions are functional rather than structural and chemical concepts. However, there is a feature that unites all bioluminescent systems—oxygen is required for all bioluminescence reactions, with no exception.

Bioluminescence investigation has a long history, and significant progress in research on the enzymes, substrates, and mechanisms of bioluminescent reactions of certain luminous organisms should certainly be acknowledged. However, it was only in the past century that advanced molecular biology techniques have provided a significant breakthrough in studying various bioluminescent systems and especially in developing novel analytical applications. The first genes encoding two-subunit luciferase from luminous bacteria *Vibrio harveyi*, firefly luciferase from *Photinus pyralis*, and Ca^2+^-regulated photoprotein aequorin from jellyfish *Aequorea victoria* were cloned almost simultaneously, probably due to the availability of these bioluminescent proteins and the highly motivated research teams involved [1,2]. Several years later, the genes encoding luciferases from marine ostracod *Vargula hilgendorfii* and soft coral *Renilla reniformis* were also cloned [1,2]. Immediately, these bioluminescent proteins were tested as reporters via the expression of genes encoding them in eukaryotic cells [3,4,5,6]. In point of fact, these studies laid the foundation for various applications of bioluminescent proteins for the in vivo imaging of intracellular events, and now, these bioluminescent technologies are widely used in all fields of biology or experimental medicine.

Later, a set of bioluminescent reporters was expanded by cloning novel luciferases from marine copepods *Metridia longa* and *Gaussia princeps* as well as decapod shrimp *Oplophorus gracilirostris* [7,8]. An interesting feature of the cloning of copepod luciferases is that the first cDNA genes encoding these proteins were isolated applying the functional screening of the relevant cDNA libraries in *Escherichia coli* by colony luminescence with a bioluminescence substrate. In this view, the natural luciferases of copepods have not been isolated or even partially characterized and seem to have no prospects to be studied. Since these luciferases demonstrated very attractive bioluminescent properties (bright bioluminescence, low molecular weight, high stability, and their own signal peptide for secretion) they were immediately and successfully applied as bioluminescent reporters for in vivo assays [8]. Nowadays, copepod luciferases, along with firefly and Renilla ones, are widely used as reporters for in vivo imaging [7,9]. The natural secreted luciferase of the *O. gracilirostris* is a tetrameric protein consisting of two monomers, each of molecular mass 19 kDa, and the two monomers of 35 kDa each [1]. The cloning of cDNA genes encoding the luciferase subunits and their following expression both in mammalian and *E. coli* cells showed that only the 19 kDa subunit revealed bioluminescent activity which appeared to be substantially less than that of the natural luciferase [10]. However, with the bioinformatic approach (the 19 kDa subunit fold turned out to bear a vague resemblance to that of the well-characterized family of intracellular lipid-binding proteins) and comprehensive mutagenesis applied, the mutant variant of the 19 kDa subunit with a higher specific activity, slower decay of bioluminescent signal, and increased solubility and thermostability was constructed. The mutant was named NanoLuc and is currently widely used as a bioluminescent reporter in various applications [9].

**Figure 1:**
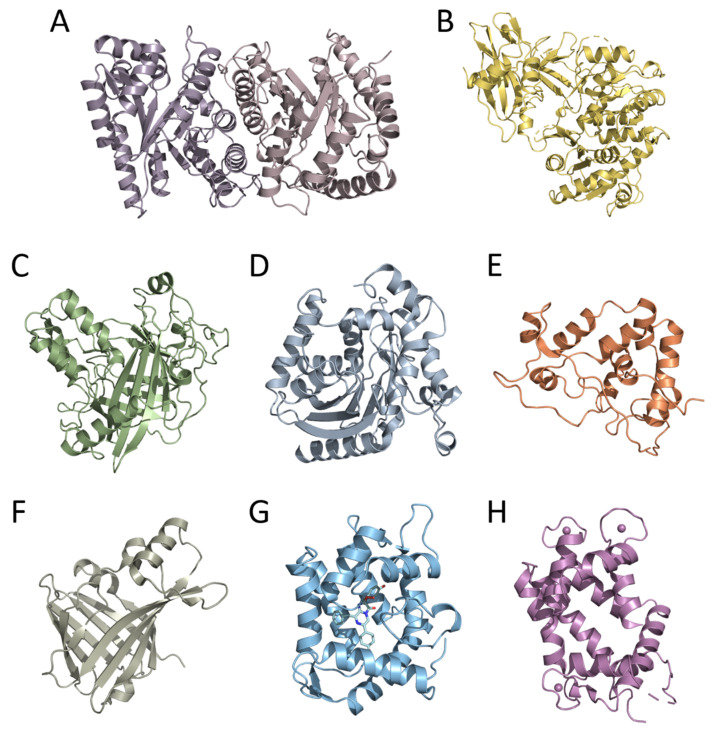
Crystal structures of bioluminescent proteins: (**A**) bacterial (PDB 1LUC), (**B**) firefly (PDB 1LCI), (**C**) dinoflagellate (PDB 1VPR), (**D**) Renilla (PDB 2PSH), (**E**) Gaussia (PDB 7D2O), and (**F**) Oplophorus (NanoLuc) (PDB 5B0U) luciferases; Ca^2+^-regulated photoproteins (**G**) aequorin (PDB 1EJ3) and (**H**) berovin (PDB 4MN0).

Moreover, the cloning of genes encoding bioluminescent proteins promoted basic research on this topic, primarily owing to the development of efficient heterologous expression systems for recombinant proteins. This allowed obtaining nearly unlimited amounts of bioluminescent proteins, thereby enabling the use of crystallography and NMR methods for the determination of their tertiary structures—a necessary step in studying the mechanism of bioluminescent reaction and function of certain amino acid residues in the catalytic oxidation of the substrate and the emitter formation. To date, the spatial structures of six luciferases and two types of Ca^2+^-regulated photoproteins have been determined. These are luciferases of luminous bacteria, firefly, dinoflagellate, soft coral, copepod, and the engineered catalytic subunit of Oplophorus luciferase (NanoLuc) as well as cnidarian and ctenophore photoproteins (Figure 1). The tertiary structures of bioluminescent proteins from organisms belonging to different taxa, as could be expected from their amino acid sequences, appeared to be completely different even in the case of Renilla, copepod, and Oplophorus luciferases that utilize the same substrate of bioluminescent reaction (coelenterazine). The only exception that has been found to date is the cnidarian and ctenophore photoproteins, which revealed the same spatial scaffold (Figure 1) despite the insignificant identity of their amino acid sequences [11]. A great variety of spatial structures and amino acid sequences of bioluminescent proteins along with the diversity of substrates and bioluminescence reaction mechanisms strongly support the conclusion that the ability to bioluminescence emerged repeatedly and independently in the course of evolution, each time adapting the available protein as a luciferase (or a photoprotein) and an appropriate organic molecule as a substrate. It may also imply that the bioluminescence function is very important for organisms and can come into being rather easily.

Site-directed and random mutagenesis is another modern technology that became applicable for research on bioluminescent proteins after genes encoding them were cloned. The application of mutagenesis was aimed both at defining the function of amino acid residues in catalysis and light-emitter formation and constructing bioluminescent reporters with improved properties. Although in many cases, the wild-type luciferases and photoproteins are “workable” for in vivo imaging in terms of their properties, there are still restricting factors for their use. These are low photon production and tissue attenuation caused by the fact that most of the wild-type bioluminescent proteins catalyze the reactions, resulting in the emission of blue-green light that is largely absorbed by mammalian tissues. This limits the sensitivity of reporting from small numbers of cells in deep-tissue locations and hinders the application of luciferases and photoproteins for imaging in animal models. The most remarkable progress has been made in improving reporter properties of firefly luciferase. Using a novel luciferin analogue (AkaLumine-HCl) and several rounds of random mutagenesis of firefly luciferase to optimize pairing the enzyme with this analogue, the novel firefly luciferase mutant named Akaluc was produced. Akaluc with the AkaLumine-HCl analogue as a substrate emitted light with a spectral maximum at 677 nm, exhibited up to a 1000-fold increase in brightness of the emission in vivo as compared to the wild-type luciferase and native luciferin, and provided single-cell detection in deep lung tissues in mice [12].

Numerous mutagenesis studies were also performed to reveal the involvement of certain amino acid residues into the catalytic reaction and generation of the excited state of the reaction product. Most of them were carried out for firefly and bacterial luciferases as well as for Ca^2+^-regulated photoproteins. Most likely, it is accounted for by the fact that the spatial structures of these proteins are also available either with a substrate analogue (firefly luciferase), a cofactor (bacterial luciferase), or with a substrate and the reaction product (Ca^2+^-regulated photoproteins). Moreover, determination of the structures of bioluminescent proteins, especially with ligands, and the results obtained on various mutants have given rise to applying computational methods for investigating the mechanisms of bioluminescent reactions. Nowadays, this valuable approach is very popular.

**Figure 2:**
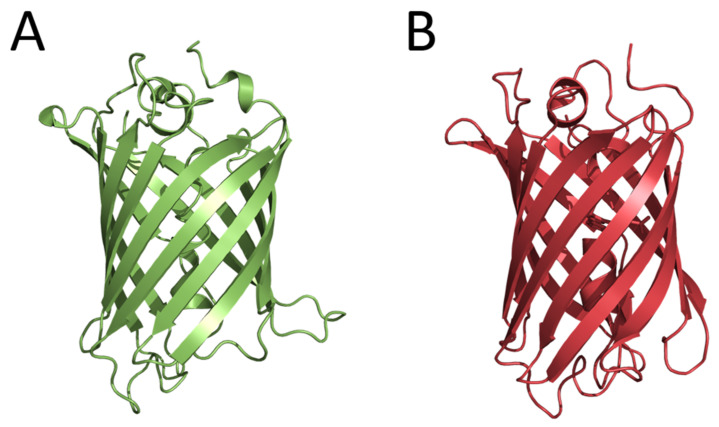
Crystal structures of wild-type Aequorea GFP (PDB 1GFL) (**A**) and GFP-like fluorescent protein DsRed (PDB 1G7K) (**B**).

The first fluorescent proteins, now widely used for in vivo imaging, were also found in luminous organisms. In 1962, the green-fluorescent protein (later named GFP) was isolated from the luminous jellyfish *A. victoria* at the purification of Ca^2+^-regulated photoprotein aequorin as a contaminant protein [13]. Although in the years to follow, the biochemical and spectral properties of GFP were studied and the structure of chromophore was determined, only in 1992 did cloning the gene encoding GFP and the demonstration that *E. coli* cells expressing the GFP gene become fluorescent [14] open up new avenues for studies, engineering, and various analytical applications of this protein. In 1994, the GFP gene was first expressed in nematode *Caenorhabditis elegans* for the purpose of imaging gene expression [15]. In effect, this research paved the way for the widespread application of fluorescent proteins for in vivo imaging of intracellular processes. At the same time, studies aimed at obtaining the GFP mutants with altered spectral characteristics were started. As a result, the cyan fluorescent protein (CFP), which could be paired with GFP for the development of FRET (Förster resonance energy transfer) methods to monitor protein–protein interactions, was constructed. These studies allowed suggesting the mechanism of the GFP chromophore formation. Considering that the determination of the tertiary structure of a protein is a key step in understanding the mechanism of its functioning, several research groups entered the competition to solve the GFP structure. The crystal structures were practically determined simultaneously for the GFP mutant and the wild-type GFP. GFP appeared to be an almost-perfect cylinder composed of 11 β-strands surrounding a helix running up the central axis, into which the chromophore was inserted (Figure 2). The chromophore was deeply buried at the center of the protein that ensured its isolation from the solvent and consequently provided its bright fluorescence. In 2008, Dr. Shimomura, Prof. Chalfie, and Prof. Tsien received the Nobel Prize in Chemistry (https://www.nobelprize.org/prizes/chemistry/2008/summary/ (accessed on 22 December 2022)) for the discovery of GFP, the contribution to its studies, and the development of in vivo imaging technologies.

In 1999, six genes encoding fluorescent proteins homologous to the GFP from *A. victoria* were cloned from non-bioluminescent reef corals. It was the first example demonstrating that GFP-like proteins are not always functionally linked to bioluminescence [16]. Two of these fluorescent proteins had yellow and red fluorescence, i.e., their fluorescence spectra drastically differed from that of GFP from *A. victoria*. It turned out that these fluorescent proteins had no amino acid sequence identities with Aequorea GFP but shared the same β-can fold characteristic of this protein (Figure 2). Later, the GFP-like fluorescent proteins were found in many marine non-luminous organisms. This important opening initiated new directions of basic research and the construction of novel fluorescent reporters for in vivo analytical applications. The fields of application of GFP and GFP-like fluorescent proteins as well as their numerous mutants in biology and experimental medicine are too diverse and numerous to be listed here.

## 2. Modern Studies and Applications of Bioluminescent and Fluorescent Proteins

The Special Issue “Bioluminescent and Fluorescent Proteins: Molecular Mechanisms and Modern Applications”, which is international in scope and diverse in content, aims to provide a multidisciplinary platform for the publication of reviews and experimental papers devoted to fundamental studies in the field, the construction of novel bioluminescent and fluorescent proteins, the development of new methods with the use of bioluminescent and fluorescent proteins, and applications of these unique proteins in various research areas. From 2020 up to now, the Special Issues dealing with various aspects of this topic have assembled eight reviews and 24 experimental articles.

All the reviews are highly informative and mostly related to modern applications of fluorescent and bioluminescent proteins for vivo and in vitro studies.

In his review, Dr. Cardarelli [17] considers and discusses the novel applications of fluorescent proteins for “dynamic” imaging of an inert tracer that, diffusing and colliding, affords quantitative information on the intracellular landscape in terms of accessibility/connectivity and obstacles/barriers to motion. The essence of such an approach is exemplified by applications of fluorescent proteins within the cytoplasm and nucleus. In addition, the author outlines the directions of future development of this valuable approach since he believes that the experimental data on molecular diffusion collected in the interior of cells may explain crucial and still-obscure phenomena, such as the biologic benefit of anomalous transport, the regulation of protein folding/unfolding, intracellular signaling, target search processes, and bimolecular reaction kinetics.

Another review concerns the application of genetically encoded sensors based on fluorescent proteins for high-throughput drug screening [18]. The authors comprehensively overview successful, prospective, and hopeful attempts to use these sensors in the high-throughput screening of modulators of ion channels, Ca^2+^ homeostasis, the activity of G-protein-coupled receptor as well as for screening of cytotoxic, anti-cancer, and anti-parasitic compounds. The described examples demonstrate that fluorescent sensors can be successfully applied in high-throughput drug screening alongside bioluminescent proteins which, nowadays, are more frequently used in these kinds of assays due to their low background signals. Moreover, the advantages of sensors in whole-organism drug screening models and the perspectives of combining human disease modeling using CRISPR techniques with genetically encoded fluorescent sensors for drug screening are also discussed.

The review by Smylla et al. [19] summarizes applications of fluorescent proteins in the Drosophila eye as part of genetic screens for studying the rhodopsin expression patterns, subcellular protein localization, membrane protein transport, and as genetically encoded biosensors for the in vivo determination of Ca^2+^ transients and phospholipids. The fluorescent proteins and sensors based on them, as well as the microscopic methods which are used in different studies on the Drosophila eye, are described here in detail. Moreover, the authors also discuss the advantages of the recently developed photoactivatable fluorescence proteins that are suitable for super-resolution or correlative light electron microscopy (CLEM) analysis. This comprehensive review is obviously useful not only for researchers who study the Drosophila eye, but also for scientists investigating other sensory or neuronal systems.

The work of Endo and Ozawa [20] focuses on bioluminescent reporters emitting red or near-infrared light that are demanded for the highly sensitive imaging of various biochemical processes in living animals. The authors review in detail the properties of firefly, Renilla, and NanoLuc luciferases and their numerous mutants as well as the effects of modifications of substrates (D-luciferin in the case of the firefly and coelenterazine for Renilla and NanoLuc luciferases). In addition, they consider the application of BRET technology for the construction of red-shifted bioluminescent reporters based on radiation-less resonance energy transfer (Förster mechanism) where bioluminescent and fluorescent proteins are the donor and acceptor, respectively. The given examples clearly show the potential and value of this approach. The review will provide support for researchers involved in the development of novel red-shifted bioluminescent reporters and for those who plan to use the reporters for the visualization of certain processes in living animals to select the most appropriate ones for their studies.

The review of Saito-Moriya et al. [21] also deals with bioluminescent reporters with red-shifted light emission. However, in contrast to the above-mentioned study [20], the authors focus on firefly luciferase mutants and artificial analogues of the reaction substrates. The combinations of luciferase mutants and artificial analogues allowed developing reporter systems with far-red light emission [12] and even providing near-infrared-wavelength bioluminescence (λ_max_ = 706–743 nm). Thus, in view of the recent remarkable achievements, firefly luciferase reporter systems appear to be highly attractive candidates for imaging in living animals. This review, like the above one [20], may be of interest both for researchers elaborating novel reporter systems and for those who use bioluminescent reporters in their studies.

The review of Krasitskaya et al. [9] covers modern biomedical in vivo and in vitro applications of coelenterazine-dependent bioluminescent proteins such as Ca^2+^-regulated photoproteins, Renilla and copepod luciferases, NanoLuc, and pholasin. These proteins originate from various luminous marine organisms and differ in amino acid sequences and tertiary structures, but utilize the same substrate, coelenterazine, for bioluminescent reaction [1,7]. The authors discuss the biochemical and bioluminescent features of wild-type proteins as well as their mutants possessing the properties appropriate their use in various in vivo and in vitro analytical assays. By providing numerous examples, they demonstrate the high analytical potential of coelenterazine-dependent bioluminescent proteins and technologies based on them (BRET, complementation assays, etc.) for biomedical research. This review may also be of interest for researchers who plan to apply coelenterazine-dependent proteins in their studies as well as for those involved in the development of novel technologies for imaging intracellular events and in vitro assays.

The review by Perin et al. [22] surveys the employment of luminous dinoflagellates in toxicity bioassays. The authors highlight previous and current achievements in studies of the bioluminescent system of these luminous organisms as well as different approaches for their use in detecting certain pollutants in marine sediment and seawater. They propose that toxicity tests based on luminous dinoflagellates may sit in the middle between fast bacterial assays and more complex toxicity tests involving vertebrates and invertebrates. This review may draw the interest of researchers who develop novel toxicity assays and those engaged in monitoring environmental pollution.

In contrast to the above-mentioned reviews, Timsit et al. [23] discuss the function of light emission in the vital activity of unicellular organisms such as bacteria and dinoflagellates. They propose that, like in visual animals, the interplay between light emission and reception may play multiple roles in intra- and interspecific communication and participate in complex behavior in the unicellular world. This hypothesis is mainly based on findings that many unicellular organisms possess a variety of photoreceptors capable of perceiving and integrating light stimuli of different wavelengths which produce responses ranging from phototaxis to more complex behaviors. The proposed hypothesis looks plausible but needs to be experimentally verified, although it is undoubtedly original. This review may interest a wide scientific readership and encourage researchers for new studies on the topic.

The experimental articles are mainly concerned with the development of new methods applying various fluorescent and bioluminescent proteins as well as the construction of reporter molecules with improved properties and the synthesis of novel firefly luciferin and coelenterazine analogues. Although all the published papers substantially contribute to the corresponding areas of research, I would like to draw the attention of readers to some of them that, in my opinion, are most interesting.

Li and Cui [24] demonstrate that through the co-expression of NanoLuc and the genetically encoded mini singlet oxygen generator (miniSOG), the light emission generated by luciferase upon substrate addition can cause activation of the cholecystokinin 1 receptor (CCK1R) by means of singlet oxygen produced by miniSOG. The authors suggest that this approach can be used in various in vivo applications.

The paper by Salgado-Almario et al. [25] describes comparative studies on the applicability of four fluorescent genetically encoded ratiometric Ca^2+^ indicators based on troponin C (TN-XXL, Twitch-1, Twitch-2B, and Twitch-4) to monitor calcium transients in the heart of zebrafish embryos. Based on the obtained results, the authors conclude that Twitch-1 and Twitch-4 may be the most promising genetically encoded ratiometric indicators for producing transgenic zebrafish lines, which could be used for modeling heart disorders, drug screening, and cardiotoxicity assessment during drug development. Since, nowadays, zebrafish is considered a cost-effective vertebrate model for many studies, the article may be of interest to a wide range of readers.

The cell-based cytotoxicity assay using human hepatocytes to estimate the effects of drug-metabolizing enzymes on cytotoxicity is reported in the article by Iwado et al. [26]. These researchers constructed human hepatoblastoma HepG2 cells expressing click beetle luciferase and major drug-metabolizing enzymes (CYP2C9, CYP2C19, CYP2D6, and CYP3A4) and used them to monitor the time-dependent CYP-mediated cytotoxicity by bioluminescence. Real-time bioluminescence measurement revealed that, as compared to CYP-non-expressing cells, the light intensity of CYP-expressing cells rapidly decreased when the cells were treated with low concentrations of aflatoxin B1 or primaquine, which exhibit cytotoxicity in the presence of CYP3A4 or CYP2D6, respectively. Using kinetics data obtained by the real-time bioluminescence measurement, the authors estimated the time-dependent changes in 50% inhibitory concentration (IC_50_) values in the aflatoxin B1- and primaquine-treated cell lines. The developed cell lines can be applied for testing the toxicity of various chemical compounds.

A description of the simultaneous detection of two genes’ expression into the soil nematode *Caenorhabditis elegans* using green- and red-emitting luciferases is presented in the paper by Doi et al. [27]. Here, the authors were able to observe the expression of two genes in a single animal from embryonic to adult stage over its whole life span. This is the first application of a two-color luciferase system to a whole animal that allows the relationship of the expression patterns of multiple genes of interest to be precisely analyzed over the whole life of the animal, dependent on the changes in genetic and/or environmental conditions. Since *C. elegans* is frequently used as a model organism in various studies, this paper may be interesting for a wide range of readers.

The results obtained by Höring et al. [28] are another example of effectively applying bioluminescent technologies for the study of G-protein-coupled receptors (GPCRs). The authors describe a novel proximal functional assay for the routine characterization of putative ligands for four histamine receptor subtypes (H1R, H2R, H3R, and H4R), applying the split-NanoLuc and the recently engineered mini g proteins. With this method applied, the functional response upon receptor activation was monitored in real time and the four mini g sensors were evaluated by investigating the selected standard agonists and antagonists. This approach is applicable for research on the functional activity of other GPCRs. Since GPCRs are the most studied drug targets and are addressed by more than 30% of approved drugs, this paper may be of interest for those conducting research in this field.

The method to detect viral infection in mice described by Gaspar et al. [29] is also based on applying split-NanoLuc technology. The small high-affinity peptide tag (HiBiT) consisting of only 11 amino acids was engineered into a clinically used oncolytic adenovirus, and the complementary large protein (LgBiT) was constitutively expressed in tumor cells. Infection of the LgBiT-expressing cells with the HiBiT oncolytic virus led to the formation of active NanoLuc in the cytosol of the cells, providing bright-light emission upon treatment with a substrate. Moreover, the use of the recently developed furimazine analogue, hydrofurimazine, of a higher aqueous solubility as compared to furimazine as a reaction substrate allowed a significant increase in light intensity and, consequently, the sensitivity of the method. The described approach could provide novel opportunities for studying the biology of viruses in animal models, which may interest the researchers studying various viral infections.

The paper by Larionova et al. [30] gives an example of using copepod luciferase for an in vitro assay. The authors constructed a hybrid protein consisting of the smallest isoform of *M. longer* luciferase (MLuc7) and 14D5a single-chain antibody to the surface glycoprotein E of tick-borne encephalitis virus as a model fusion partner. They showed that, whereas the fusion of a single-chain antibody to either the *N*- or *C*-terminus of MLuc7 does not affect the luciferase bioluminescence properties, the binding site of the single-chain antibody influences its binding capacity—the affinity of the 14D5a-MLuc7 hybrid protein where the *C*-terminus of the single-chain antibody was fused to the *N*-terminus of MLuc7 appeared to exceed that of MLuc7-14D5a by 2.5-fold. The 14D5a-MLuc7 hybrid protein was tested both in a model immunoassay and to detect the virus in tick extracts. Noteworthy is that, although the properties of copepod luciferases such as excellent thermal stability and bright luminescence are very suitable to use as labels in various in vitro bioluminescence assays, the examples of their applications are few, which is mainly caused by difficulties in producing the correctly folded recombinant copepod luciferases in inexpensive expression systems [8].

The next two articles [31,32] are dedicated to improvement in near-infrared imaging technology based on a firefly-type bioluminescent system [12]. The paper by Viviani et al. [31] describes the construction and characterization of the mutant of PxRE beetle luciferase which, in combination with the 6′-(1-pyrrolidinyl) luciferin analogue, provided the highest bioluminescent activity with a spectral maximum at 650 nm and a catalytic efficiency ~2.5 times higher than that in combination with native firefly luciferin. Moreover, this combination provided light signals several orders of magnitude higher than that of the commercial AkaLumine with firefly luciferase, a slower bioluminescence decay time, and 3-fold higher activity of bioluminescence in vivo in bacterial cells as compared to firefly luciferin, owing to the better penetrability of the 6′-(1-pyrrolidinyl) luciferin analogue across bacterial cell membranes. The authors believe that the PxRE beetle luciferase mutant combined with the 6′-(1-pyrrolidinyl) luciferin analogue is a highly promising reporter for bioimaging in mammalian tissues. The article by Nakayama et al. [32] also concerns the improvement in near-infrared imaging technology. Although the use of AkaLumine-AkaLuc for imaging [12] allows light detection at the single-cell level, there is a problem in monitoring weak luminescence signals from micrometastases near the liver surface due to the high hepatic background signal. In this case, the authors suggest using the synthesized seMpai luciferin analogue which provides a high bioluminescent signal comparable with the AkaLumine analogue, but, in contrast, it allows detecting weak light signals from micrometastases due to the lack of any background signal. It was proposed that obtaining novel firefly luciferase mutants against the seMpai analogue with higher activity may allow the high-sensitivity detection of micrometastases in live animal models.

The article by Subach and co-authors [33] presents the construction of blue-to-red fluorescent timer (mRubyFT) from the bright mRuby2 red fluorescent protein. It was demonstrated that the mRubyFT blue form reached its maximum in 5.7 h and completely transformed into the red form with a maturation half-time of 15 h. The blue and red forms of purified mRubyFT were correspondingly 4.1-fold brighter and 1.3-fold dimmer than those of the mCherry-derived Fast-FT timer in vitro, but both of them turned out to be 1.3-fold brighter than the corresponding forms of Fast-FT. Since mRubyFT is monomeric, this allowed the labeling and confocal imaging of cytoskeleton proteins in live mammalian cells. In addition, the X-ray structure of the red form of mRubyFT at 1.5 Å resolution was determined. To determine the impact of the residues surrounding the chromophore on the mRubyFT timer properties, the set of mutants was also produced and characterized. The results described may interest researchers both involved in the development of novel fluorescent reporter proteins and those applying fluorescent reporter proteins for imaging intracellular processes.

The paper by Bakayan et al. [34] reports on the creation of Ca^2+^ sensors based on Redquorin fusion protein of Ca^2+^-regulated photoprotein aequorin with tdTomato fluorescent protein with enhanced sensitivity to calcium ions. Redquorin is spectrally optimal for deep-tissue in vivo imaging due to a large red shift of the emission spectrum (λ_max_ = 582 nm), but has low sensitivity to Ca^2+^ which limits its usefulness as a sensor of intracellular Ca^2+^ signals. A total of twenty-four amino acid positions that belong to EF-hand domains and their surroundings in the spatial structure of apo-aequorin were selected by the authors for mutagenesis. As a result of screening six aequorin single or double mutants (AequorinXS) with mutations localized in the EF-hand domains 2 and 3, and the C-terminal tail with a markedly higher Ca^2+^ sensitivity as compared to that of wild-type aequorin were isolated. All corresponding Redquorin mutants showed higher Ca^2+^ sensitivity than that of wild-type Redquorin, and four of them (RedquorinXS) matched the sensitivity to Ca^2+^ of the GFP-aequorin sensor. Upon stable expression in the mammalian cell line, all RedquorinXS mutants reported the activation of the P2Y2 receptor by ATP with higher sensitivity than wt-Redquorin, and one of them, RedquorinXS-Q159T, outperformed the GFP-aequorin sensor. Moreover, wide-field bioluminescence imaging in mouse neocortical slices showed that RedquorinXS-Q159T and the GFP-aequorin sensor similarly reported neuronal network activities elicited by the removal of extracellular Mg^2+^. It was proposed that the constructed RedquorinXS-Q159T, a red-light-emitting Ca^2+^ sensor, is suitable for monitoring intracellular signaling in a variety of applications in cells and tissues, and is a promising candidate for the transcranial monitoring of brain activities in living mice.

Two papers are concerned with novel synthetic analogues of coelenterazine [35,36]. The latter, as is known, is a substrate of light emission reactions catalyzing the bioluminescent proteins from various marine organisms [7]. The article by Eremeeva et al. [35] describes the properties of Ca^2+^-regulated photoproteins obelin and aequorin activated by analogues with modifications of C-2, C-6, and C-8 substituents of the coelenterazine molecule. The semi-synthetic aequorins and obelins were investigated for the specific bioluminescence activity, light emission spectra, stopped-flow kinetics, and sensitivity to calcium. Several semi-synthetic photoproteins displayed sufficient bioluminescence activities accompanied by various changes in the spectral and kinetic properties as well as in calcium sensitivity. Noteworthy is that the authors found that in most cases, the semi-synthetic obelins and aequorins displayed different properties upon being activated by the same coelenterazine analogue despite the fact that the amino acid residues forming the substrate-binding pockets of these photoproteins are practically identical. The paper by Kamiya et al. [36] also deals with the synthesis and characterization of novel coelenterazine analogues with modifications of C-6 and C-8 substituents. However, in contrast to the above-mentioned study, these analogues were tested as substrates of reactions catalyzed by coelenterazine-dependent luciferases. The four coelenterazine analogues of the nine synthesized and tested were shown to generate light with completely distinctive spectral signatures and intensity patterns according to each luciferase used: Renilla luciferase, NanoLuc, and artificial luciferase (ALuc). Moreover, the quantitative properties of the spectral signatures and intensity patterns were characterized with individual or multiple reporter luciferases. The usefulness of this approach was demonstrated by applying two single-chain molecular strain probes embedding RLuc8 and ALuc23. Here, the authors suggest that this study provides a unique methodology on how to construct a bioluminescence-signature-imaging system for specifying coelenterazine-dependent marine luciferases by using their unique spectral characteristics and intensity patterns.

The article by Reyes et al. [37] is completely different in that the two non-competing technologies to diagnose urinary tract infection, TuBETUr (tube bioluminescence extinction technology urine) and CUBET (cellphone-based urine bioluminescence extinction technology), are both based on the “blackout” phenomenon that results from the exposure of luminous bacteria to an infected urine sample. Whereas TuBETUr involves active luminous bacteria and can be used in a laboratory environment, CUBET implies the reconstitution of lyophilized luminous bacteria and can be applied in a point-of-care setting. Both assays are able to detect microbes associated with urinary tract infection at less than 10^5^ CFU/mL, which is usually the lower cut-off limit for a positive urinary tract infection diagnosis. This article may be of interest not only for those who develop analytical assays but also physicians interested in applying inexpensive express diagnostic methods of urinary tract infections.

## 3. Conclusions

In summary, this short review of papers published in the Special Issue “Bioluminescent and Fluorescent Proteins: Molecular Mechanisms and Modern Applications” clearly demonstrates a variety of methods that use bioluminescent and fluorescent proteins as well as the ongoing studies aimed at improving the reporter properties of these proteins. It is beyond all doubt that the pioneering works of the last twenty years of the 20^th^ century on cloning genes encoding these proteins paved the way for these studies and applications of bioluminescent and fluorescent proteins possible.
